# Improvement of GaN-Based Device Performance by Plasma-Enhanced Chemical Vapor Deposition (PECVD) Directly Preparing h-BN with Excellent Thermal Management Characteristics

**DOI:** 10.3390/molecules30061307

**Published:** 2025-03-14

**Authors:** Yi Peng, Lingyun Liu, Qingfeng Xu, Yuqiang Luo, Jianzhi Bai, Xifeng Xie, Huanbing Wei, Wenwang Wei, Kai Xiao, Wenhong Sun

**Affiliations:** 1 Research Center for Optoelectronic Materials and Devices, School of Physical Science & Technology, Guangxi University, Nanning 530004, China; 2022070431@hzxy.edu.cn (W.W.); xkgxu2020@163.com (K.X.); 2 College of Electric Power Engineering, Guangxi Vocational College of Water Resources and Electric Power, Nanning 530023, China; liulingyun09@outlook.com (L.L.); xqf9805@163.com (Q.X.); lyq0129best@163.com (Y.L.); nnbjz11@163.com (J.B.); xiexifeng1027@yeah.net (X.X.); 15907857076@163.com (H.W.); 3 Guangxi Key Laboratory of Calcium Carbonate Resources Comprehensive Utilization, College of Materials and Chemical Engineering, Hezhou University, Hezhou 542899, China; 4 School of Chemistry and Chemical Engineering, Sichuan Institute of Arts and Science, Dazhou 635000, China; 5 State Key Laboratory of Featured Metal Materials and Life-Cycle Safety for Composite Structures, Guangxi University, Nanning 530004, China; 6 Third Generation Semiconductor Industry Research Institute, Guangxi University, Nanning 530004, China

**Keywords:** GaN based, PECVD, chemical active site, h-BN/GaN heterostructure, heat dissipation

## Abstract

As the demand for high voltage levels and fast charging rates in the electric power industry increases, the third-generation semiconductor materials typified by GaN with a wide bandgap and high electron mobility have become a central material in technological development. Nonetheless, thermal management challenges have persistently been a critical barrier to the extensive adoption of gallium-nitride-based devices. The integration of two-dimensional materials into GaN-based applications stands out as a significant strategy for tackling heat-dissipation problems. However, the direct preparation of two-dimensional materials on gallium nitride is rather challenging. In this study, high-quality h-BN was prepared directly on GaN films using plasma-enhanced chemical vapor deposition, which revealed that the introduction of appropriately sized active sites is key to the growth of h-BN. Owing to the high in-plane thermal conductivity of h-BN, the thermal conductivity of the sample has been enhanced from 218 W·m^−1^ K^−1^ to 743 W·m^−1^ K^−1^. Ultraviolet photodetectors were constructed based on the obtained h-BN/GaN heterostructure and maintained excellent detection performance under high-temperature conditions, with detectivity and responsivity at 200 °C of 2.26 × 10^13^ Jones and 1712.4 mA/W, respectively. This study presents innovative concepts and provides a foundation for improving the heat-dissipation capabilities of GaN-based devices, thereby promoting their broader application.

## 1. Introduction

Third-generation semiconductor materials represented by gallium nitride (GaN) and silicon carbide (SiC) have become the core solid-state light sources for power electronics and microwave devices due to their superior properties such as a large bandgap width, high breakdown electric field, large thermal conductivity, high electron saturation drift velocity, and strong radiation resistance [1,2,3,4]. Nevertheless, as power electronic devices are subjected to increasingly higher voltage levels and as specific high-temperature application scenarios become more prevalent, the self-heating effect resulting from substantial currents and the high-temperature environment has necessitated more rigorous thermal management for GaN-based devices [5,6,7]. The integration of two-dimensional thin film layers into micro–nano-devices has been established as a pivotal approach for significantly improving the heat-dissipation capabilities, exemplified by materials such as graphene and hexagonal boron nitride (h-BN) [8,9,10,11]. Notably, hexagonal boron nitride has garnered substantial attention in the micro–nano-device thermal management domain in recent years, owing to its relatively superior mechanical strength and chemical stability [12,13,14].

The preparation process of two-dimensional materials plays a crucial role in determining the thermal management performance of devices [15,16,17]. The prevailing approach to synthesizing h-BN involves chemical vapor deposition on a catalytic metal substrate, succeeded by mechanical exfoliation and subsequent transfer to a target substrate [18,19]. However, this technique inherently introduces contaminants, which can result in heightened thermal resistance at the interfaces of the multilayered structures. Although there have been attempts to directly prepare h-BN on the target material, the control over its crystal quality and orientation is still not perfect, or only homogeneous epitaxial growth on pre-existing h-BN templates can be achieved [20,21].

To advance the integration of h-BN with functional materials like GaN, the development of controllable methods for the direct growth of high-quality h-BN on GaN requires in-depth exploration. In this study, we demonstrate the feasibility of synthesizing high-quality h-BN on wurtzite GaN substrates via plasma-enhanced chemical vapor deposition (PECVD), leveraging their shared hexagonal crystalline structures. High-resolution characterization reveals strong alignment between the h-BN (002) and GaN (001) crystal planes. The formation mechanism of high-quality h-BN was systematically investigated by optimizing key PECVD parameters, particularly temperature and RF power, which govern plasma-induced chemical activation on GaN surfaces. Furthermore, we demonstrate thermally stable heterojunction photodetectors utilizing h-BN’s superior thermal conductivity to enhance GaN-based device performance under elevated temperatures. These findings open new possibilities for h-BN/GaN integrated applications, particularly in addressing critical challenges such as self-heating issues in GaN HEMTs.

## 2. Materials and Methods

**Figure 1:**
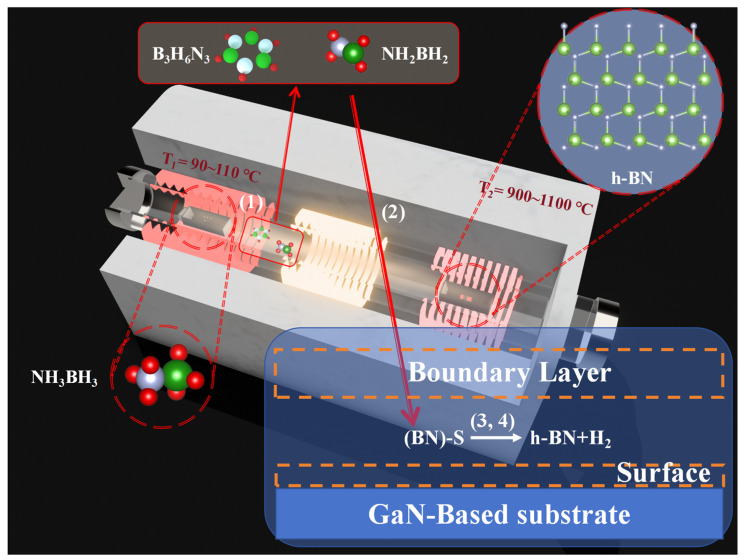
Schematic diagram of the PECVD equipment used for synthesizing h-BN and chemical reactions, (1)–(4) represent the four steps in the growth process of h-BN.

The PECVD (HF-Kejing, Hefei, China) apparatus employed in this study is shown in Figure 1, comprising two heating zones characterized by distinct temperature ranges and a radio-frequency (13.56 MHz) plasma generation area between them. The solid precursor, ammonia borane (NH_3_BH_3_) (Merck KGaA, Darmstadt, Germany), is positioned at the central location of the first heating zone with temperature T_1_. As the temperature T_1_ increases to a range of 90–110 °C, the sublimated precursor is transported by a carrier gas of N_2_ (40 sccm) and diffuses towards the second heating zone with temperature T_2_. The synthesis of h-BN is conducted on a substrate located at the T_2_ heating zone. The substrate employed for the deposition of h-BN is cultivated through the application of metal–organic chemical vapor deposition (MOCVD) techniques with a 4-micrometer-thick GaN epitaxial layer (Wuxi Jingdian Semiconductor Materials Co., Ltd., Wuxi, China) grown on a patterned sapphire substrate (PSS). The GaN epitaxial layer was n-type doped with silicon, achieving a carrier concentration of 1 × 10^18^ cm^−3^. Prior to synthesizing h-BN, the GaN (002) substrate was meticulously ultrasonically cleaned in a sequence of acetone, anhydrous ethanol, and deionized water, each for a duration of 20 min. The substrate was subsequently dried using nitrogen (N_2_). The growth process of h-BN in this device can be divided into four steps [22]: (1) Solid ammonia borane (NH_3_BH_3_) sublimes at 90–110 °C and is carried into the reaction zone by N_2_ at 40 sccm. Upstream of the second temperature zone (the inlet section below the growth temperature), the sublimated ammonia borane is thermally decomposed into H_2_, aminoborane (NH_2_BH_2_), and borazine (B_3_H_6_N_3_). (2) NH_2_BH_2_ and B_3_H_6_N_3_ diffuse to the chemical reaction sites on the substrate surface in the form of gas, where the expression S in Figure 1 represents the chemical active sites. (3) After these precursor molecules are adsorbed on the surface, they undergo dehydrogenation to become active substances at temperatures ranging from 700 to 1100 °C. The mixture of these active substances then interacts with the chemical sites on the GaN substrate, causing h-BN to begin nucleating on it. (4) As the reaction progresses, the h-BN thin film also acts as a barrier to the diffusion of active substances to the chemical sites on the substrate.

## 3. Results and Discussion

**Figure 2:**
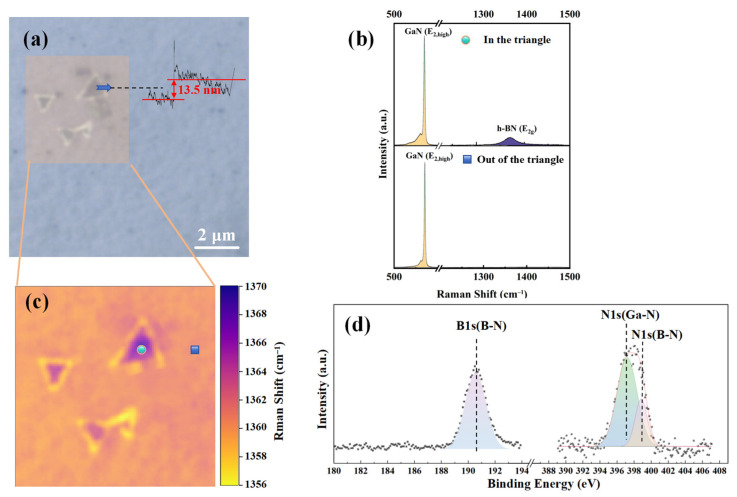
h-BN/GaN prepared by PECVD: (**a**) optical imaging at ×100 magnification; (**b**) Raman test comparison between point in the triangular morphology area and point out of the triangular area; (**c**) Raman mapping of h-BN E_2g_ phonon mode within a 5 × 5 μm^2^ area; (**d**) binding energy of B_1s_ and N_1s_ orbital in XPS.

Figure 2 shows the comprehensive characterization of the prepared h-BN. Figure 2a is the ×100 optical image of h-BN, which shows some regular triangular regions on the surface of gallium nitride, which are similar to the typical AA’ stacking configuration of h-BN [23]. The thickness of the triangle in Figure 2a can be determined to be approximately 13.5 nm by the step profiler (Bruker Corporation, Billerica, MA, USA). Figure 2c shows the Raman (Horiba Jobin Yvon, Paris, France) mapping results of the h-BN E_2g_ phonon mode [24] within a 5 × 5 μm^2^ area. Figure 2b presents the Raman results within the triangular region and outside the triangular region from Figure 2c, conducted under 532 nm laser excitation and the $Z(X,-)\bar{Z}$ Raman configuration. Both points exhibit GaN’s E_2_ and A_1_(LO) phonon modes [25]. However, only the triangular region can observe the E_2g_ phonon mode of h-BN. Figure 2d displays the high-resolution X-ray photoelectron spectroscopy (XPS) (Thermo Fisher Scientific, Waltham, MA, USA) results of the sample. The binding energy of the B_1s_ orbital is approximately 190.5 eV, and that of the N_1s_ orbital is approximately 398.1 eV, consistent with results from other studies on h-BN [26]. All of these characterization results confirm that the triangular regions are due to h-BN.

To further reveal the mechanism of h-BN ordered arrangement growth with controllable crystal orientation on GaN, multiple condition sets were established for the reaction temperature zone (T_2_ temperature zone), radio-frequency power, and growth time. The sample numbers and corresponding growth conditions are shown in Table 1.

**Figure 3:**
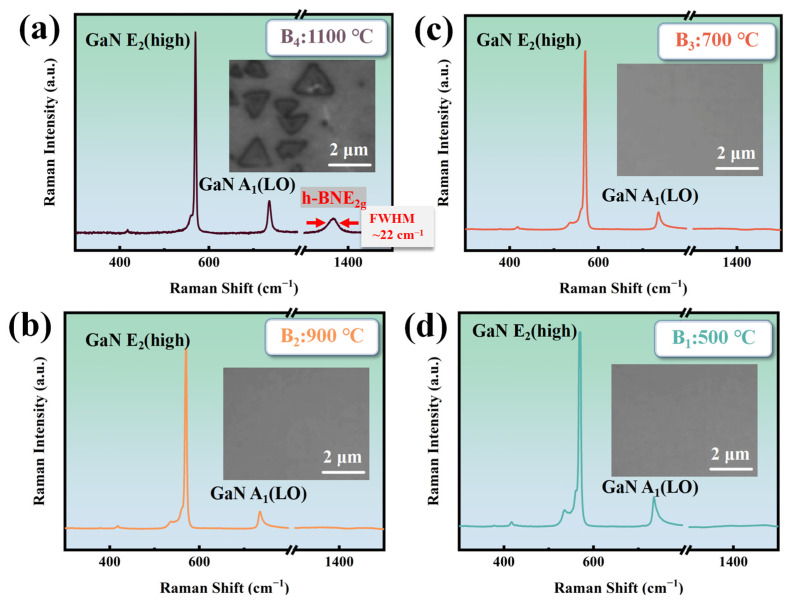
(**a**) The Raman spectrum and morphology of B_4_ at 1100 °C (RF power: 0 W; deposition time: 30 min); (**b**) the Raman spectrum and morphology of B_3_ at 900 °C (RF power: 0 W; deposition time: 30 min); (**c**) the Raman spectrum and morphology of B_2_ at 700 °C (RF power: 0 W; deposition time: 30 min); (**d**) the Raman spectrum and morphology of B_1_ at 500 °C (RF power: 0 W; deposition time: 30 min).

Figure 3 shows the Raman spectroscopy and surface morphology of the B_1_~B_4_ sample The B_4_ sample has a Raman FWHM (full width at half maximum) of the E_2g_ phonon mode of about 22 cm^−1^, indicating the good crystalline quality. This is attributed to the use of a single precursor source with a nitrogen-to-boron ratio of 1:1, which ensures synchronized release of stoichiometrically equivalent B and N atoms during thermal decomposition. This intrinsic stoichiometric fidelity guarantees precise control over the B/N ratio in h-BN, effectively circumventing elemental imbalance caused by disparate reaction kinetics in conventional dual-precursor approaches [27]. The Raman spectroscopy and surface morphology of the B_1_~B_4_ in Figure 3b–d indicate that the growth temperature of h-BN on GaN needs to reach 1100 °C without plasma assistance. Based on the literature research, GaN starts to decompose at temperatures exceeding 1100 °C [28], creating areas of high chemical reactivity. This phenomenon is a key factor in the crystallization of h-BN on the GaN surface.

**Figure 4:**
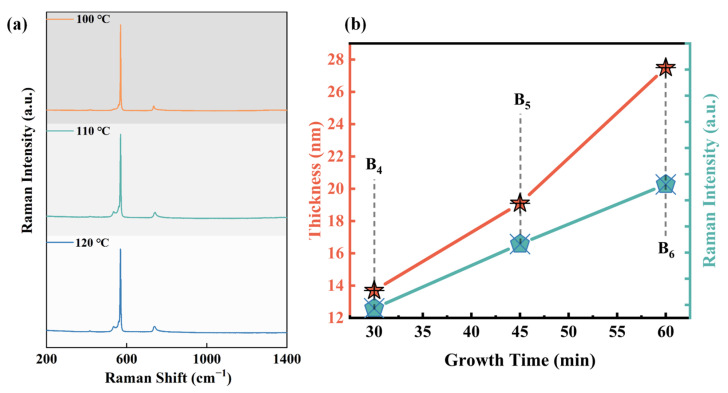
(**a**) Raman comparison of samples with different preheating temperatures; (**b**) dependence of the h-BN thickness and Raman intensity on the growth time.

In addition to the sedimentation temperature of the main reaction regulating sample preparation, the temperature of the preheating reaction zone used for sublimating solid ammonia borane precursors also needs to be considered. At room temperature, ammonia borane, which serves as the precursor, exists in the form of solid crystals and melts at around 106 °C [29]. Figure 4a shows the Raman results at three different preheating temperatures of 100 °C, 110 °C, and 120 °C (with the main reaction temperature being 1100 °C). Surprisingly, the Raman signal for h-BN can not to be detected in any of the samples. In fact, previous studies have shown that thermal decomposition of borazine can occur within the temperature range of 77–137 °C [29], and under appropriate conditions, borazine can completely decompose below its melting temperature. Setting the preheating temperature too high can cause ammonia borane to melt violently at the beginning of the reaction, which is not conducive to control of the rate and can affect the quality of the film deposition in the main reaction zone. Therefore, all subsequent experiments were conducted with the preheating temperature set at 90 °C.

Figure 4b also illustrates the thickness controllability of the h-BN preparation process. The graph shows the change in Raman intensity and h-BN growth thickness with deposition time from 30 min to 60 min at a growth temperature of 1100 °C, corresponding to samples B_4_, B_5_, and B_6_. The results indicate that both Raman intensity and deposition thickness exhibit good linearity with time, demonstrating that this growth method can stably and conveniently control the deposition thickness of h-BN by adjustment of the deposition time, with a growth rate of about 28 nm/h.

**Figure 5:**
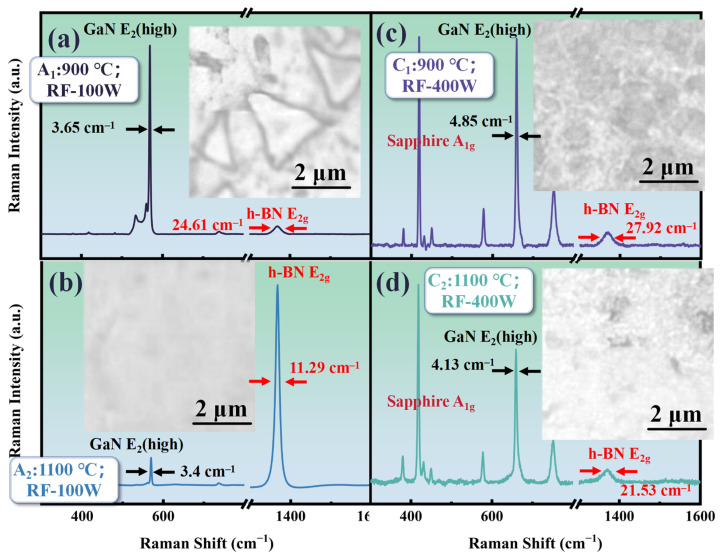
(**a**) The Raman spectrum and morphology of A_1_ (T: 900 °C; RF: 100 W; t: 30 min); (**b**) the Raman spectrum and morphology of A_2_ (T: 1100 °C; RF: 100 W; t: 30 min); (**c**) the Raman spectrum and morphology of C_1_ (T: 900 °C; RF: 400 W; t: 30 min); (**d**) the Raman spectrum and morphology of C_2_ (T: 1100 °C; RF: 400 W; t: 30 min).

Although h-BN can be prepared on GaN without the use of plasma assistance by increasing the temperature, prolonged exposure to high temperatures may lead to excessive decomposition of the GaN layer, affecting the crystal quality. Therefore, it is extremely necessary to study the addition of plasma assistance to reduce the reaction temperature or improve the crystal quality of h-BN. Figure 5 shows the Raman and morphology comparison of samples A_1_ (T: 900 °C; RF: 100 W; t: 30 min), A_2_ (T: 1100 °C; RF: 100 W; t: 30 min), C_1_ (T: 900 °C; RF: 400 W; t: 30 min), and C_2_ (T: 1100 °C; RF: 400 W; t: 30 min). As is shown in Figure 5, the E_2g_ Raman peak of h-BN can also be measured in the A_1_ sample prepared at 900 °C after increasing the RF power of the plasma assistance to 100 W. The triangular morphology of h-BN can be observed in Figure 5a. The triangular morphology of h-BN obtained at 1100 °C has larger sizes compared to those without plasma assistance.

Ammonia borane can undergo dehydrogenation at temperatures ranging from 700 to 1100 °C. In theory, this precursor could be used for chemical vapor deposition of h-BN at these temperatures, but it was found that temperatures need to reach 1100 °C for deposition on GaN. It should be noted that in the reaction flowchart for preparing h-BN from ammonia borane, as shown in Figure 1, the third step indicates that the active mixture produced by the precursor needs to interact with chemically active sites on the substrate to initiate h-BN nucleation. The surface activity of dielectric materials is generally lower compared to catalytic metals. After bombardment with high-energy plasma, the number of active sites on the material surface can be increased, which is why the addition of radio-frequency plasma allows h-BN deposition at relatively lower temperatures. Moreover, from Figure 5a, it can be seen that the FWHM of the E_2g_ Raman peak for h-BN in the A_1_ sample is about 24 cm^−1^, which is close to the result at 1100 °C without plasma assistance (about 22 cm^−1^).

In Figure 5a, the h-BN E_2g_ Raman peak signal intensity of the A_2_ sample is significantly enhanced. By combining this with the morphology image, it can be seen that, unlike the scattered distribution of the triangular morphology without plasma assistance, after increasing the RF plasma excitation power to 100 W, the h-BN deposited at a temperature of 1100 °C shows a uniform distribution over a larger area on GaN. Moreover, from Figure 5b, it can be obtained that the FWHM of the h-BN E_2g_ Raman peak in the A_2_ sample is further reduced to about 12 cm^−1^, which is much smaller compared to the FWHM (about 35 cm^−1^) of h-BN directly grown on other dielectric materials [30].

To further investigate the regulation of RF power on the deposition of h-BN on GaN using PECVD, we increased the RF power to 400 W. However, as is shown in Figure 5c,d, when the RF power further increased, the Raman spectra of samples C_1_ and C_2_ show a trend that the signal from the sapphire substrate becomes dominant, and the Raman characteristic peaks of GaN and h-BN both exhibit attenuation. Moreover, compared to the samples at 100 W under the same temperature conditions, the FWHM of the Raman characteristic peaks of GaN and h-BN has broadened. This may be caused by the following reasons: when the RF power is further increased, the density of the plasma cloud in the reaction chamber significantly increases; meanwhile, excessive energy can cause plasma bombardment on the GaN surface and etch the GaN layer to some extent, thereby increasing structural disorder [30].

In the corresponding morphology image of Figure 5c,d, the pits observed on the sample surface at 900 °C and the holes at 1100 °C under 400 W RF power are both evidence of GaN surface etching. The difference is that h-BN has a relatively fast growth rate at 1100 °C, meaning that it grows and merges on GaN, and finally forms holes in the pits.

**Figure 6:**
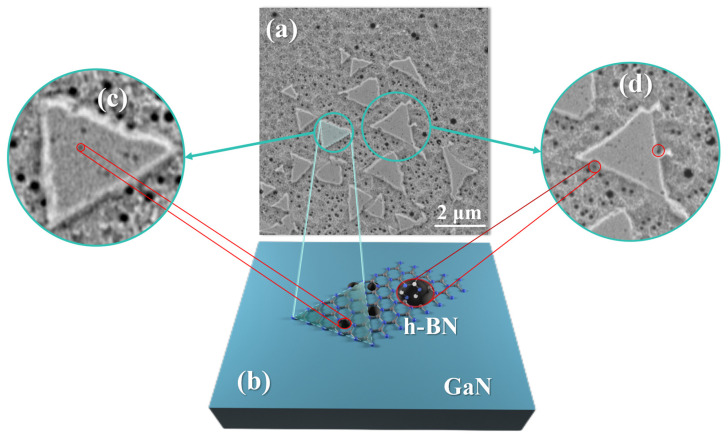
(**a**) SEM morphology image of A_1_ sample; (**b**) temperature and plasma cause corrosion pit on the GaN surface, which can be used as the active site of growing h-BN; (**c**) small corrosion pit facilitates the growth of h-BN; (**d**) large corrosion pit hinders h-BN merging.

The importance of appropriate RF power and temperature can be better illustrated by the SEM (Carl Zeiss AG, Oberkochen, Germany) morphology of the A_1_ sample shown in Figure 6. Figure 6a indicates that under the combined effects of plasma and temperature, many active etch pits appear on the GaN surface, and the triangular morphology of h-BN mainly grows on the small etch pits. Figure 6c is an enlarged image, where h-BN can be seen growing on the surface with stronger activity near the small etch pits and then merging. However, when the plasma RF power is increased, the size of the etch pits on the GaN surface becomes larger, resulting in h-BN appearing as is shown at point B in Figure 6d. The h-BN layers are subjected to greater stress at this point, causing film tearing, which leads to the holes in the h-BN film, as shown in Figure 5c,d, and a decrease in uniformity.

**Figure 7:**
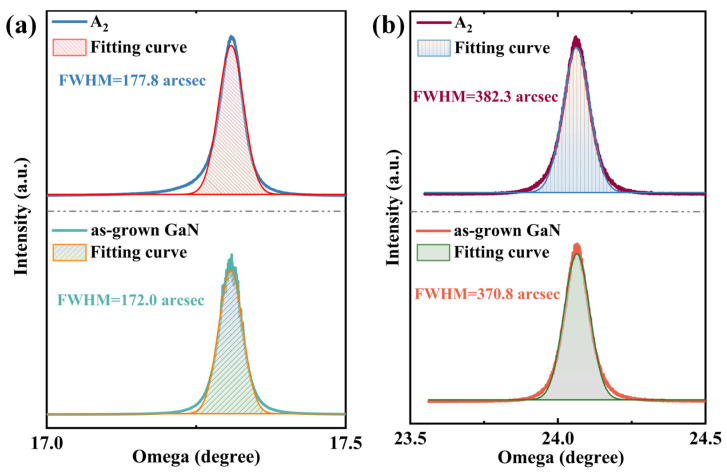
Comparison of (**a**) (002) and (**b**) (102) high-resolution X-ray diffraction rocking curves of the A_2_ sample and as-grown GaN.

With the introduction of RF plasma assistance, the growth quality of h-BN was improved and the growth temperature was reduced. However, the regulation of the GaN layer by RF plasma also needs attention to ensure that GaN does not experience a significant decline in quality, which is a prerequisite for effectively combining h-BN with GaN. Figure 7 shows the XRD (Malvern Panalytical, Malvern, United Kingdom) rocking curve test results for the GaN layer of the A_2_ sample. The results indicate that after the growth of h-BN by PECVD, the FWHM of the GaN epitaxial layer’s (002) rocking curve widened from 172.0 arcsec to 177.8 arcsec, and the FWHM of (102) widened from 370.8 arcsec to 382.3 arcsec. The introduction of plasma caused some corrosion pits in GaN, which inevitably led to a certain degree of quality deterioration. However, due to the activity provided by the plasma and the 1:1 nitrogen–boron ratio of the ammonia borane precursor, the reaction time can be further shortened to ensure that the quality of GaN does not significantly decline.

The epitaxial relationship of heterojunctions also has a significant impact on the performance of materials and devices. To better protect the GaN layer and reduce the growth process time, the A_3_ (T: 1100 °C, RF: 100 W, t: 5 min) sample was selected. The epitaxial relationship and crystal orientation of h-BN and GaN were further studied using the HRTEM method.

**Figure 8:**
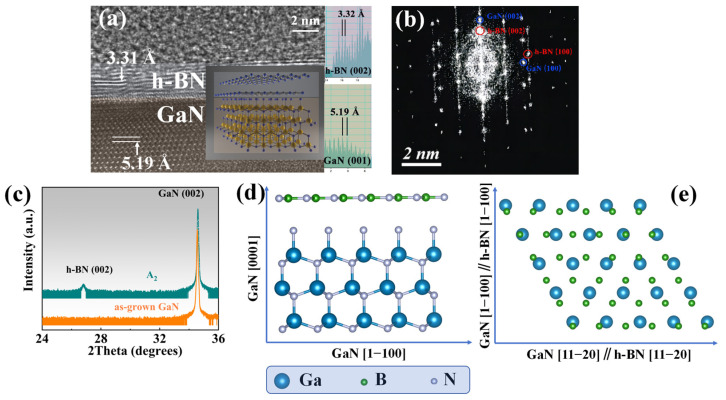
(**a**) The HRTEM image of sample A_3_ (T: 1100 °C, RF: 100 W, t: 5 min) under the deflected zone axis, showing an obvious double-layer structure. The insert shows the measured crystal plane spacing of the crystal planes parallel to the sample surface corresponding to each layer; (**b**) the FFT image of the sample A_3_ under the zone axis [11−20]; (**c**) the HRXRD 2θ&ω scan curve of the A_1_ sample and as-grown GaN; (**d**) cross-sectional view of the epitaxial relationship between h-BN and GaN in the sample A_3_; (**e**) top view of the epitaxial relationship between h-BN and GaN in the sample A_3_, where GaN [1−100]//h-BN [1−100] and GaN [1−120]//h-BN [1−120].

Figure 8a shows the HRTEM (Thermo Fisher Scientific, Waltham, MA, USA) image of the A_3_ sample under the [11−20] zone axis, where the layer structure distribution of the sample can be clearly distinguished. Double layers can be observed in the image, and the inset measures the interplanar spacing parallel to the sample surface of the three-layer structure. The interplanar spacing of the topmost layer is 3.32 Å, corresponding to the (002) plane of h-BN, with a total thickness of about 2~3 nm, corresponding to 7~10 layers of h-BN. The bottom layer is the GaN layer, and the interplanar spacing of the (001) plane is 5.19 Å, which is also measured in the inset.

By measuring the various crystal planes and combining the results of the FFT diffraction pattern in the Figure 8b, the epitaxial relationship of h-BN grown on GaN was determined, that is, h-BN (002) parallel to GaN (001) and the sample surface, as shown in Figure 8d. GaN [1−100] parallel to h-BN [1−100] and GaN [1−120] parallel to h-BN [1−120] are shown in Figure 8e. The epitaxial relationship of h-BN and GaN observed in HRTEM was proven over a larger range by the HRXRD 2θ&ω scan shown in Figure 8c. Among them, h-BN (002) is parallel to GaN (002) and sapphire (006), and it should be noted that the samples selected for this process are A_1_, as the h-BN layer in A_3_ samples is too thin to be detected by HRXRD.

After demonstrating the fabrication of high-quality h-BN directly on GaN using PECVD, an analysis was conducted to study the improvement in thermal management characteristics of h-BN/GaN materials. Thermal management performance is primarily characterized by measuring the thermal conductivity. Due to the small thickness of GaN and h-BN thin films, conventional methods for measuring thermal conductivity (such as the laser flash method) require larger sample sizes and thicknesses, which are not suitable for thin films. Photothermal Raman testing is a rapid, non-destructive method for measuring thermal conductivity in thin films, utilizing the strong temperature dependence of Raman modes and the ability of the Raman laser to serve as a localized heat source [31].

In cylindrical coordinates, the local temperature distribution caused by laser irradiation on a material can be represented by Equation (1) [32]:

(1) $$T(r,z)=\frac{\alpha AP}{2\pi K}\int_{0}^{\infty }J_{0}(\lambda \frac{r}{r_{0}})exp(-\frac{\lambda^{2}}{4})\times \frac{\alpha r_{0}exp(-\lambda \frac{z}{r_{0}})-\lambda exp(-\alpha z)}{{\left(\alpha r_{0}\right)}^{2}-\lambda^{2}}d\lambda$$

where *λ* is the variable of the first-kind Bessel function, *J*_0_(*λr*) is the zeroth-order first-kind Bessel function, *K* is the thermal conductivity, *A* is the absorbance, *α* is the absorption coefficient, *P* is the laser power, and *r*_0_ is the radius of the laser spot.

**Figure 9:**
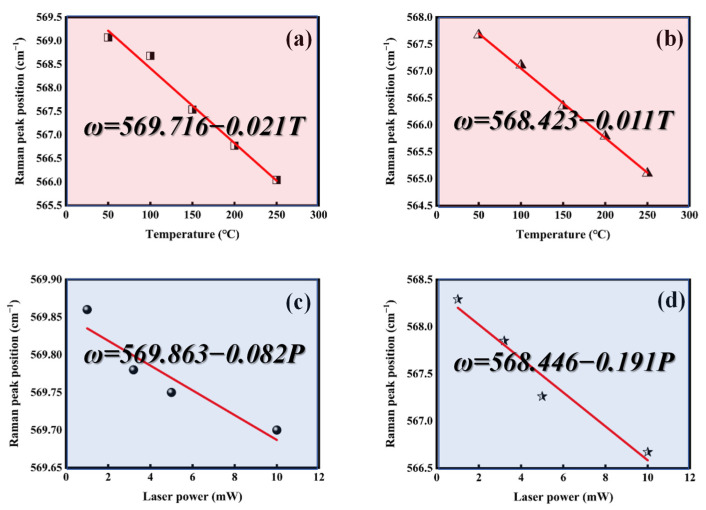
Temperature (*T*) dependence of the Raman peak position (*ω*) measured for (**a**) A_2_ (*dω*/*dT*) and (**b**) GaN (*dω*/*dT*), and power (*P*) dependence of the Raman peak position (*ω*) measured for (**c**) A_2_ (*dω*/*dP*) and (**d**) GaN (*dω*/*dP*).

After simplification through integration calculations, the following is derived:

(2) $$K=\frac{\alpha A\bar{N}(r,z)dP}{V_{c}d\bar{T}}=\frac{\alpha A\bar{N}(r,z)(d\omega /d\bar{T})}{V_{c}(d\omega /dP)}$$

where *V_c_* is the laser detection volume, $\bar{N}(r,z)$ is the average temperature factor, *dω*/*dT* is the temperature coefficient (the rate of change in the Raman peak with average temperature), and *dω*/*dP* is the power coefficient (the rate of change in the Raman peak with excitation power). It should be noted that in tests measuring the Raman peak variation with average temperature, to minimize the effects of laser heating, a light source that is not easily absorbed by the material should be used. The bandgaps of GaN and h-BN are 3.4 eV and 5.9 eV, respectively, corresponding to intrinsic absorption wavelengths of 362 nm and 210 nm. Therefore, a 532 nm laser was chosen as the excitation source for variable temperature tests. In contrast, during tests measuring the Raman peak variation with power, the thermal effect of the laser is utilized, and thus a 325 nm laser was selected as the excitation source. Additionally, for the selection of target peaks in Raman testing, the GaN E_2_(high) peak was chosen in this study, which is most sensitive to temperature, and the result is shown in Figure 9.

The thermal conductivity increased from 218 W·m^−1^ K^−1^ of the original GaN to 743 W·m^−1^ K^−1^ of the sample after the deposition of h-BN (A_2_ sample), as calculated by formula 2. The results are summarized in Table 2.

Table 3 shows the thermal conductivity of samples A_1_, A_2_, C_1_, and C_2_ calculated through Raman photothermal testing, indicating that the uniformity of the surface morphology has a significant impact on the thermal conductivity.

To investigate the effect of enhanced thermal management on the performance of devices, a photodetector designed for the detection of ultraviolet light was created The device fabrication was based on sample A_2_. The removal of byproducts generated during the PECVD process was performed sequentially as follows:

1. Ultrasonic cleaning in deionized water twice (each for 10 min);
2. Rapid thermal annealing (RTA) treatment at 500 °C for 10 min under a N_2_ atmosphere;
3. Ultrasonic cleaning in isopropanol and deionized water (10 min each), followed by drying with N_2_ blowing.

**Figure 10:**
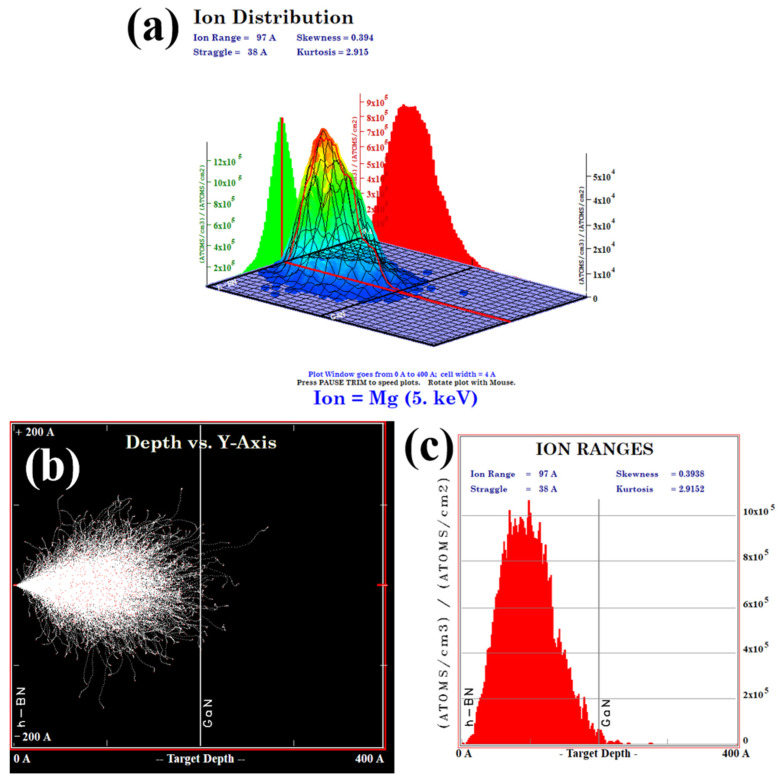
(**a**) The 3D diagram of the injected ion distribution obtained from the TRIM software (version: SRIM-2013) simulation; (**b**) the graph of the injection depth of Mg^2+^ ions obtained from simulation in 20 nm h-BN/4 μm n-GaN; (**c**) the 2D diagram of the injected ion distribution.

**Figure 11:**
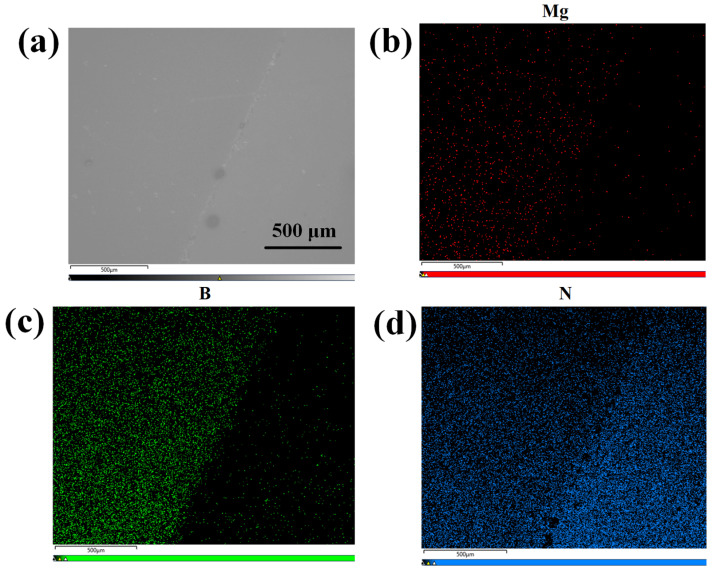
(**a**) The surface SEM image at 40× magnification of h-BN:Mg/n-GaN; (**b**) EDS map corresponding to Mg element in h-BN:Mg/n-GaN; (**c**) EDS map corresponding to B element in h-BN:Mg/n-GaN; (**d**) EDS map corresponding to N element in h-BN:Mg/n-GaN.

Subsequently, ion implantation was employed to dope the h-BN layer, followed by post-doping RTA treatment at 1000 °C for 10 min under a N_2_ atmosphere. The implantation energy was set to 5 keV with Mg^2+^ as the ion source, the implantation dose was optimized to 1 × 10^15^ ions/cm^2^ to achieve uniform doping. As is demonstrated by the TRIM (Transport of Ions in Matter) simulation in Figure 10, this energy selection ensures that the ion implantation does not affect the underlying GaN layer. The effectiveness of h-BN doping is conclusively demonstrated by the Energy-Dispersive X-ray Spectroscopy (EDS) (Carl Zeiss AG, Oberkochen, Germany) results for the Mg, B, and N elements, as shown in Figure 11.

**Figure 12:**
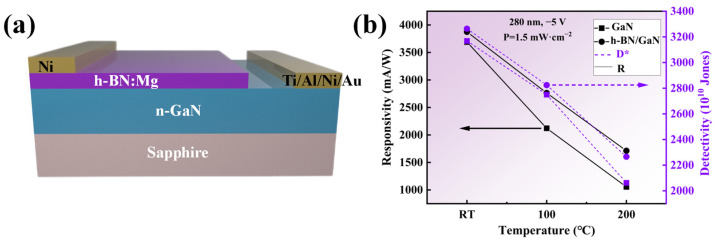
(**a**) Schematic illustration of the photoresponse of the h-BN:Mg/n-GaN heterojunction device. (**b**) Responsivity and detectivity as a function of temperature under −5 V bias voltages under illumination of 1.5 mW/cm^2^ for 280 nm.

Figure 12 shows the utilization of a heterostructure of h-BN:Mg/n-GaN, as depicted in Figure 12a. Electrode materials consisting of Ni and Ti/Al/Ni/Au, with a thickness of 100 nm, were deposited on the h-BN and GaN layers using a magnetron sputtering technique, respectively. Following the electrode deposition, the samples were subjected to a 5 min rapid thermal annealing process at 600 °C within a nitrogen-protected environment inside a rapid annealing furnace. A commercial UV-LED with an emission wavelength of 280 nm and optical power density of 1.5 mW/cm^2^ was used as the ultraviolet light source. The key parameters of the detector, namely responsivity and detectivity, can be determined by Equations (3) and (4), respectively [33]:

(3) $$R=\frac{I_{L}-I_{D}}{P_{i}S}=\frac{I_{P}}{P_{i}S}$$

where *I_L_* is the current under UV light illumination, *I_D_* is the dark current, *I_P_* is the photocurrent, *P_i_* is the power density of the incident light, and *S* is the effective photoresponse area (0.2 cm^2^ for this experiment).

(4) $$D^{*}=\frac{\sqrt{S}\cdot R}{\sqrt{2qI_{D}}}$$

where *q* is the charge constant, and *I_D_* is the dark current of the device.

Figure 12b shows the performance comparison of photodetectors based on h-BN/GaN and those based on GaN under the same illumination and bias voltage conditions at different temperatures, the current-voltage characteristics of the device and the time-dependence of the photocurrent were measured with a semiconductor parameter analyzer (Tektronix, Beaverton, OR, USA). Under room-temperature conditions, the specific detectivity and responsivity of the photodetector based on h-BN/GaN are 3.26 × 10^13^ Jones and 3875.8 mA/W, respectively, which are 3.0% and 5.0% higher than those based on GaN photodetectors. This is due to the coupling of the p–n junction and the heterojunction with a large bandgap difference in the h-BN/GaN structure, which enhances the built-in electric field and thus the rectification effect, resulting in a reduced dark current. According to Formulas (3) and (4), the detectivity and responsivity increase. At high temperatures, the advantages of the thermal management characteristics provided by h-BN become apparent. When the temperature reaches 200 °C, the detectivity and responsivity of the GaN-based photodetector decrease by 34.9% and 71.4%, respectively, compared to those at room temperature, while for the h-BN/GaN-based photodetector, these values are 30% and 55.8%, respectively, verifying that the excellent thermal management characteristics provided by h-BN are beneficial for improving the heat-dissipation performance of GaN devices.

Table 4 shows the performance comparison of the current study with previous studies on the application of h-BN and GaN in photodetectors. This study has shown improvements in thermal conductivity, detectivity, and responsivity.

## 4. Conclusions

In summary, this study primarily aims to improve the performance of GaN-based devices at high temperatures by utilizing the heat-dissipation properties of two-dimensional h-BN. It explores a method to directly deposit h-BN on GaN using PECVD and investigates the effects of different process parameters, as well as the ultimate enhancement of device performance. SEM tests revealed that the chemical active sites introduced by plasma on GaN are key to directly preparing h-BN. The thermal conductivity of GaN increased from 218 W m^−1^ K^−1^ to 743 W m^−1^ K^−1^ after the preparation of h-BN. Photodetectors based on the h-BN/GaN structure exhibited more-stable performance at high temperatures compared to those made of pure GaN. At an environmental temperature of 200 °C, the detectivity and responsivity of GaN photodetectors decreased by 34.9% and 71.4%, respectively, compared to room-temperature conditions, while these data for h-BN/GaN photodetectors were 30% and 55.8%, respectively. Future research will further explore methods to break through the thickness limitations of high-quality h-BN, with the aim of further enhancing the performance of devices. These results indicate that using PECVD is an effective method for preparing high-quality h-BN on GaN and can provide new solutions to address the self-heating effect in GaN-based devices.

## Figures and Tables

**Table 1:** Different process conditions of PECVD method for direct preparation of h-BN on GaN.

Sample	Deposition Temperature (°C)	RF Power (W)	Deposition Time (Min)
A_1_	900	100	30
A_2_	1100	100	30
A_3_	1100	100	5
B_1_	500	0	30
B_2_	700	0	30
B_3_	900	0	30
B_4_	1100	0	30
B_5_	1100	0	45
B_6_	1100	0	60
C_1_	900	400	30
C_2_	1100	400	30

**Table 2:** The average temperature coefficients, the power coefficients of Raman peak position and thermal conductivity for the GaN and A_2_.

Sample	dω/dT (cm^−1^/°C) E_2_ (High) (GaN)	dω/dP (cm^−1^/mW) E_2_ (High) (GaN)	Thermal Conductivity K (W·m^−1^ K^−1^)
GaN	0.011	0.191	218
A_2_	0.021	0.082	743

**Table 3:** The thermal conductivity comparison of h-BN/GaN samples at different deposition temperatures and RF powers.

Sample	A_1_	A_2_	C_1_	C_2_
Thermal Conductivity K (W·m^−1^ K^−1^)	369	743	201	432

**Table 4:** The average temperature coefficients, the power coefficients of Raman peak position, and thermal conductivity for GaN and h-BN/GaN.

References	Structure	Thermal Conductivity K (W·m^−1^ K^−1^)	Detectivity D* (Jones)	Responsivity R (mA/W)
Lu et al. *Nanotechnology* **2016**, *27*, 48LT03 [34]	ZnO quantum dot-doped graphene/ h-BN/GaN	/	1.0 × 10^13^ (248 nm)	1915 (248 nm)
Peng et al. *Nanomaterials* **2023**, *13*, 1546 [35]	h-BN (hole):/ GaN:Si	425	2.6 × 10^13^ (280 nm)	1970.7 (280 nm)
This study	h-BN:Mg/ GaN:Si	743	3.2 × 10^13^ (280 nm)	3875.8 (280 nm)

## Data Availability

The data presented in this study are available on request from the corresponding authors.
